# Comparative Genomics of *Erwinia amylovora* and Related *Erwinia* Species—What do We Learn?

**DOI:** 10.3390/genes2030627

**Published:** 2011-09-15

**Authors:** Youfu Zhao, Mingsheng Qi

**Affiliations:** Department of Crop Sciences, University of Illinois at Urbana-Champaign, 1201 W. Gregory Dr., Urbana, IL 61801, USA; E-Mail: msqi@illinois.edu

**Keywords:** comparative genomics, *in silico* subtractive hybridization, type III secretion, effector, exopolysaccharide amylovoran, virulence, fire blight, apple, pear, *Erwinia pyrifoliae*

## Abstract

*Erwinia amylovora*, the causal agent of fire blight disease of apples and pears, is one of the most important plant bacterial pathogens with worldwide economic significance. Recent reports on the complete or draft genome sequences of four species in the genus *Erwinia*, including *E. amylovora*, *E. pyrifoliae*, *E. tasmaniensis*, and *E. billingiae*, have provided us near complete genetic information about this pathogen and its closely-related species. This review describes *in silico* subtractive hybridization-based comparative genomic analyses of eight genomes currently available, and highlights what we have learned from these comparative analyses, as well as genetic and functional genomic studies. Sequence analyses reinforce the assumption that *E. amylovora* is a relatively homogeneous species and support the current classification scheme of *E. amylovora* and its related species. The potential evolutionary origin of these *Erwinia* species is also proposed. The current understanding of the pathogen, its virulence mechanism and host specificity from genome sequencing data is summarized. Future research directions are also suggested.

## 1. Introduction

Fire blight, caused by the gram-negative bacterium *Erwinia amylovora*, is the first plant bacterial disease confirmed back in the 1880s and is a devastating necrotic disease affecting apples, pears and other rosaceous plants [1]. Currently, the disease is widespread across North America, Europe and the Middle East including Iran, threatening the native origin of apple germplasm resources in Central Asia. Although more than two centuries have passed and significant progress has been made in revealing the mysteries of the pathogen and the disease, many questions remain unanswered. Most notable ones are questions regarding the pathogen, its ability to cause disease, and interaction with host plants and insect vectors. Why natural isolates of *E. amylovora* display differential virulence? What are the molecular mechanisms underlying the host specificity of *Erwinia* strains, as some have wide host range, whereas others with limited host range? What are the genetic differences between them? In this review, we summarize the current understanding of pathogen from genome sequencing efforts in four *Erwinia* species, and highlight what we have learned from comparative genomic analyses, as well as genetic and functional genomic studies. Future perspectives on research for this important pathogen are also suggested.

## 2. *Erwinia amylovora* and Related *Erwinia* Species

As a member of the *Enterobacteriaceae*, *E. amylovora* is a gram negative rod-shaped bacterium, which is related to many important human and animal pathogens such as *Escherichia coli*, *Salmonella enterica*, *Shigella flexerni*, and *Yersinia pestis*. *E. amylovora* is capable of infecting various hosts within the family of Rosaceae including subfamily Spiraeoideae. However, some *Erwinia* strains are host-specific, which can only infect *Rubus* plants within the subfamily of Rosoideae. Furthermore, differential virulence among strains isolated from Spiraeoideae has been demonstrated on different apple cultivars [2,3]. These observations and earlier genetic studies have divided *E. amylovora* strains into three major groups with different host range; *i.e.*, strains isolated from Spiraeoideae, from Rosoideae (*Rubus* spp.), and from Asian pear (a new species *E. pyrifoliae*). *E. pyrifoliae*, the causal agent of bacterial shoot blight disease of Asian pears, is only reported in Japan and South Korea [4]. In Spain, another species *E. piriflorinigrans* has been confirmed to cause necrosis of pear blossoms [5]. Other related *Erwinia* species are *E. tasmaniensis* and *E. billingiae*, both saprophytic microorganisms isolated from apple blossoms in Australia and trees in UK, respectively [6,7]. Knowledge of new *Erwinia* species has brought new challenges for management of fire blight disease, especially for international trade regulation, which has been greatly hindered due to insufficient information regarding *E. amylovora* and related *Erwinia* species.

This situation could change greatly as we find out more about the genetic composition of these microorganisms. Currently, genomes of four species from the genus *Erwinia*, including three *E. amylovora* strains, three *E. pyrifoliae* strains (two from Korean and one from Japan), and one *E. tasmaniensis* and *E. billingiae* strain each, have been sequenced and published [6-12] (Table 1). These genome sequences might have provided us near complete genetic information about *E. amylovora* and its closely-related species. Comparative genomic studies thus could be conducted to determine the relatedness and evolution of genes/proteins within the genomes of these closely related *Erwinia* species.

**Table 1:** Overview of genome sequencing of *Erwinia amylovora* and related species.

**Strains[Reference]**	**Size (Mb)**	**G+C content**	**Total proteins**	**Plasmid #s**	**Host**	**Sequencing methods**
*E. amylovora* CFBP1430 [11]	3.81	53.6	3706	1	*Crataegus*	Illumina
*E. amylovora* ATCC 49946 [10]	3.81	53.6	3565	2	Apple	Sanger
*E. amylovora* BAA2158 [9]	3.81	53.6	3857	3	Blackberry	454
*E. pyrifoliae* 1/96 [6]	4.03	53.4	3697	4	Asian pear	454/Sanger
*E. pyrifoliae* DSM 12163 [12]	4.03	53.4	4038	4	Asian pear	454/Illumina
*E. pyrifoliae* Ejp617 [8]	3.91	53.6	3672	5	Asian pear	454
*E. tasmaniensis* Et1/99 [7]	3.88	53.7	3622	5	Apple flower	Sanger
*E. billingiae* Eb661 [6]	5.1	55.2	4917	2	Tree	454/Sanger

## 3. Comparative Genomics of *Erwinia amylovora* and Related *Erwinia* Species

The genomes of *E. amylovora* and its related species range from 3.8 to 5.1 Mbp (Table 1), with *E. amylovora* contains the smallest genome compared to other enterobacteria sequenced so far (up to 5.5 Mbp) [13]. A comparison of genomes of *E. amylovora* strains CFBP1430 (isolated from *Crataegus* in France) and ATCC 49946 (also called Ea273, an apple isolate from New York) shows that the two genomes share more than 99.99% identity at the nucleotide level, indicating that *E. amylovora* is a relatively homogeneous species as indicated previously [11,14]. However, due to annotation differences, the total predicted proteins in ATCC 49946 and CFBP1430 are 3565 and 3706, respectively, without considering that the latter does not contain the pEA72 plasmid [9,11]. The genomes of the two *E. pyrifoliae* strains from Korea (Ep1/96 and DSM 12163 (Ep16/99) are almost identical [14]. The same problem exists with genome annotation for *E. pyrifoliae* strains, in which 3697 and 4038 proteins in Ep1/96 and DSM 12163, respectively, are predicted [6,12]. In order to simplify our comparison, discussion described below is mostly based on annotations of CFBP1430 and DSM 12163 genomic data except otherwise mentioned.

**Figure 1:**
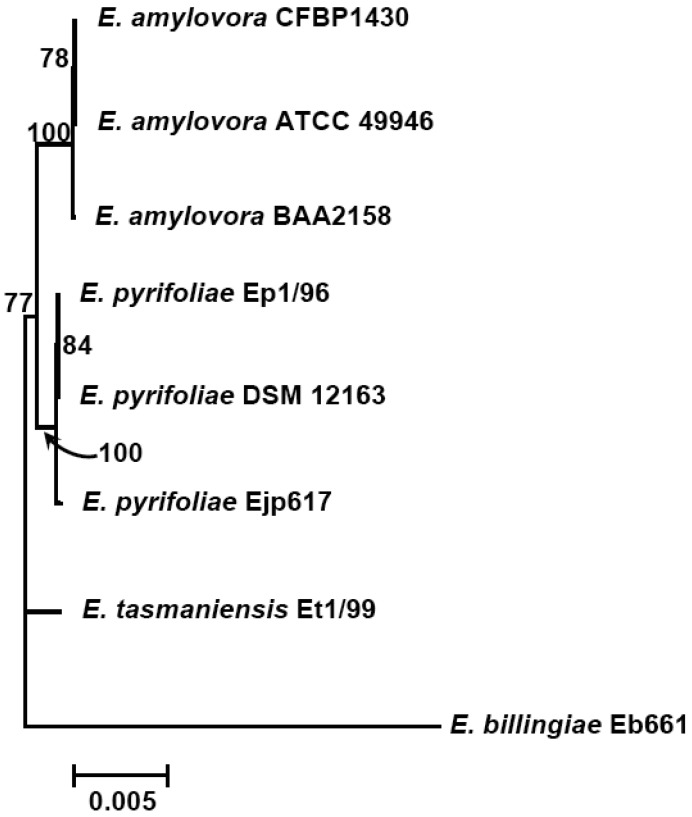
Evolutionary relationship of *Erwinia amylovora* and related *Erwinia* species. Phylogenetic tree was constructed from concatenated sequences (2222 aa) of four housekeeping proteins (AcnB, GltA, GyrB and RpoD) using Neighbor-Joining (NJ) method. Bootstrap scores greater than 60 are given at each node. The scale bar represents 0.005 amino acids substitutions per site. *E. billingiae* strain Eb661 was used as an outgroup.

The subtractive hybridization-based mGenomeSubtractor program is used to run BLAST searches of the reference genome against one or multiple bacterial genomes as reported recently for *in silico* comparative genomic analyses [15,16]. Table 2 shows the numbers of specific and conserved proteins for each *Erwinia* genome against five others. Specific and conserved proteins are arbitrarily defined for those proteins with homology (H) value less than 0.42 and more than 0.81, respectively [15,16]. The most significant conclusion from this table is that the number of conserved proteins is around 2100, no matter which genome as reference, indicating these 2100 proteins may represent the “core” proteins of *E. amylovora* and related *Erwinia* species. In contrast, the specific proteins vary among genomes, indicating these proteins are unique ones for each genome. A phylogenetic tree reflecting their potential evolutionary relationship is thus generated using conserved housekeeping proteins (Figure 1).

**Table 2:** Numbers of conserved and specific proteins in *Erwinia* genomes compared to five related *Erwinia* genomes. Specific and conserved proteins are arbitrarily defined for those proteins with H value less than 0.42 and more than 0.81, respectively. The genomes included in the comparison are *E. amylovora* strain CFBP1430, ATCC BAA2158, *E. pyrifoliae* DSM 12163, Ejp617, *E. tasmaniensis* strain Et1/99, and *E. billingiae* strain Eb661. In case of *E. amylovora* strain ATCC 49946 and *E. pyrifoliae* strain 1/96, the genomes of *E. amylovora* strain CFBP1430 and *E. pyrifoliae* strain DSM12163 are not included in the comparison, respectively.

**Genome compared to five genomes**	**Total proteins**	**Specific proteins**	**Conserved proteins**	**Intermediate**
*E. amylovora* CFBP1430	3706	147	2122	1437
*E. amylovora* ATCC 49946	3565	268	2124	1173
*E. amylovora* BAA2158	3857	217	2122	1518
*E. pyrifoliae* DSM 12163	4038	502	2149	1387
*E. pyrifoliae* 1/96	3697	282	2153	1262
*E. pyrifoliae* Ejp617	3672	204	2145	1323
*E. tasmaniensis* Et1/99	3622	588	2108	926
*E. billingiae* Eb661	4917	1954	2072	891

**Figure 2:**
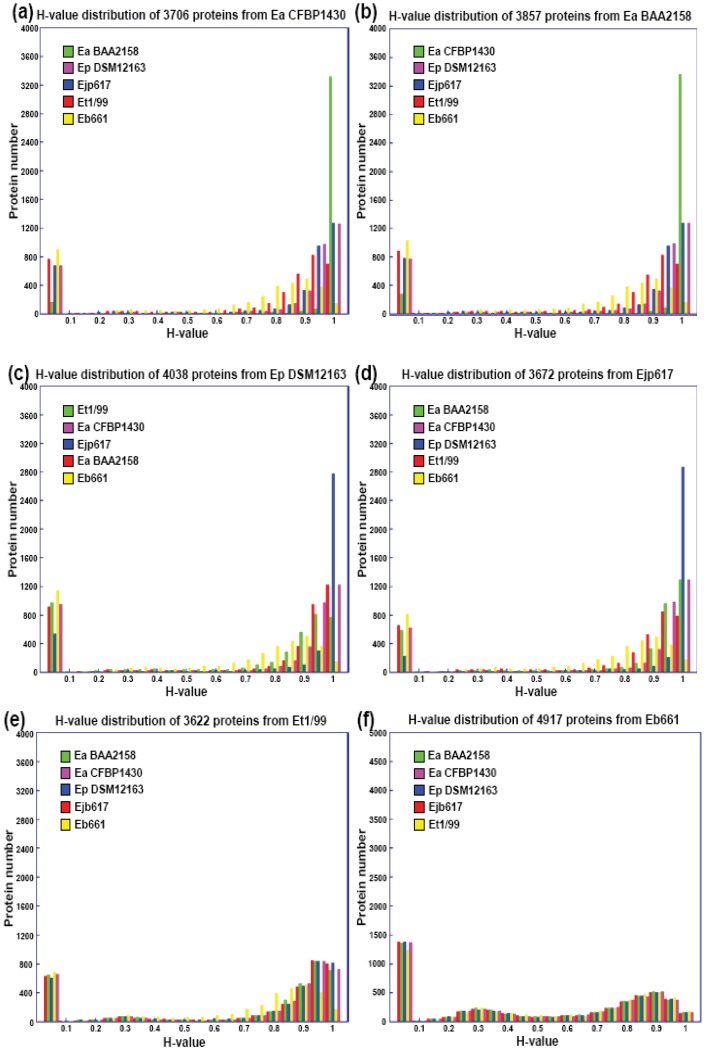
Histogram of BLASTP-based homology value (H value) distribution of predicted proteins in *Erwinia* strains compared to five sequenced related *Erwinia* genomes. (**a**) *E. amylovora* strain CFBP1430; (**b**) *E. amylovora* strain ATCC BAA2158; (**c**) *E. pyrifoliae* DSM 12163; (**d**) *E. pyrifoliae* Ejp617; (**e**) *E. tasmaniensis* strain Et1/99; (**f**) *E. billingiae* strain Eb661. The H-value reflects the degree of similarity in terms of length of match and the degree of identity at amino acid level between the matching protein in the subject genome and the query genomes with E value <10^−8^. Specific and conserved proteins are arbitrarily defined as previously described [15] for those genes with H value less than 0.42 and more than 0.81, respectively. Other proteins are defined with H values between 0.42 and 0.81.

Further analyses indicate that the majority of conserved proteins in *Erwinia* species have H-values of more than 0.85, except *E. billingae*, which representing the core proteins among them and suggesting erwinias are evolutionally conserved (Figures 1 and 2). On the other hand, the majority of specific proteins in erwinias have H-values of less than 0.1, except *E. billingae* (Figure 2). Interestingly, when compared *E. amylovora* strains CFBP1430 or ATCC 49946 to BAA2158, a strain with limited host *Rubus*, more than 3400 of 3500 conserved proteins (98%) have H-values of 1 (Figure 2(a,b), Table 3), indicating the genomes of *E. amylovora* strains are almost identical, no matter their host range or geographic origin. These results further indicate that not much has been changed for genomes of *E. amylovora* except several recombination events [11] since the disease spread from North America to Europe about sixty years ago [1]. A similar conclusion could also be drawn for strains of *E. pyrifoliae* from Japan and Korea, in which about 85% conserved proteins (2800 out of 3300) have H-values of 1 (Figure 2 (c,d), Table 3). When compared *E. amylovora* strains with those of *E. pyrifoliae* strains, they share about 2800 conserved proteins (Table 3). However, the number of proteins with H-values of 1 drops dramatically to around 1200 (Figure 2), indicating diversification occurs for these two pathogenic species and suggesting these two species may be evolutionally derived from two separate sources, one in North America and the other in Asia (Figure 1). On the other hand, these results reinforce the assumption that *E. amylovora* and *E. pyrifoliae* are both relatively homogeneous species and further support the current classification scheme of *E. amylovora* and *E. pyrifoliae* as separate species, though they cause similar disease.

In contrast, the two saprophytic *Erwinia* species are distantly related to those pathogenic species (Figure 1), as indicated by the number of conserved proteins and the conservativeness of those proteins (Figure 2 (e,f), Table 3). Among them, *E. tasmaniensis* is more closely related to pathogenic *Erwinia* species than that of *E. billingae* (Figure 1). *E. tasmaniensis* and *E. billingae* share about 2600 and 2200 conserved proteins with pathogenic *Erwinia* species, respectively (Table 3). However, the number of proteins with H-values of 1 further drops, along with H-value below 1 increases dramatically, especially for those with H-value between 0.42 and 0.85 (Figure 2 (e,f), Table 3), indicating more diversification or changes occur in these saprophytic microorganisms and suggesting diversification may be related to its free-living life style or originated from different evolution sources than those of pathogenic ones.

On the other hand, the majority of specific proteins among *Erwinia* species have H-value of 0 (Figure 2), indicating these proteins are indeed unique proteins presented in the *Erwinia* genomes. As reported in pseudomonads [16], many specific proteins in erwinias except *E. billingae* are plasmid-borne, indicating that acquisition and maintenance of plasmids may represent a major mechanism for erwinias to change their genetic composition, which may represent a major mechanism of bacterial genome evolution [17].

**Table 3:** Numbers of conserved and specific proteins in *Erwinia* genomes compared to five related *Erwinia* genomes individually. Specific and conserved proteins are arbitrarily defined for those proteins with H value less than 0.42 and more than 0.81, respectively. CP: conserved proteins; SP: specific proteins. Ea: *E. amylovora*; Ep: *E. pyrifoliae*; Et: *E. tasmaniensis;* Eb: *E. billingiae*. Numbers in each row indicate the specific and conserved proteins presented in genome of the corresponding subject genome (first column) as compared to query genomes (head row).

**Comparison of genomes**	**Ea** **CFBP1430**		**Ea** **BAA2158**		**Ep** **DSM 12163**		**Ep** **Ejp617**		**Et** **Et1/99**		**Eb** **Eb661**	
	CP	SP	CP	SP	CP	SP	CP	SP	CP	SP	CP	SP
CFBP1430	---	---	3495	175	2831	802	2826	803	2678	910	2278	1134
BAA2158	3526	297	---	---	2858	905	2851	910	2680	1034	2270	1274
DSM 12163	2864	1086	2863	1061	---	---	3349	618	2731	1164	2293	1427
Ejp617	2838	745	2842	722	3302	292	---	---	2706	832	2296	1076
Et1/99	2648	879	2628	891	2659	849	2627	876	---	---	2344	988
Eb661	2276	2253	2262	2254	2239	2283	2245	2279	2371	2125	---	---

## 4. Pathogenicity and Host Specificity of *Erwinia amylovora*: What is Known and What Remains Unknown?

*E. amylovora* has been developed as a model pathogen in studying plant-microbe interactions since the first cell free elicitor (HrpN, Harpin) was identified in 1992 [18,19]. The production of a functional hypersensitive response and pathogenicity (*hrp*)-type III secretion system (T3SS) and the exopolysaccharide amylovoran in *E. amylovora* are strictly required for inciting disease on host plants. Recent studies suggest that they are two major, yet separate virulence factors [20]. A T3SS island deletion mutant and an *ams* operon deletion mutant could complement each other in a co-inoculation experiment, indicating that a functional T3SS and the amylovoran are both necessary, but can be supplied by distinct bacterial strains outside of bacterial cells to cause disease [20].

The majority of *hrp* T3SS genes are encoded on the pathogenicity island 1 (PAI1). The T3SS system of *E. amylovora* secretes virulence effector proteins, including HrpA, HrpN, HrpW, and disease-specific protein DspE/A [21-23]. Many studies, including genome sequencing, have reached the conclusion that only five effector genes (*eop1*, *eop3*, *avrRpt2*_Ea_, *dspA/E*, and *hopC1*) exist in the genome of *E. amylovora*, which are subject to direct *hrpL* regulation, a master regulator of T3SS [23-25]. *DspA/E*, *avrRpt2*_Ea_, and *hopC1* have been demonstrated to be induced in immature pear fruit, indicating that they may play a major role in virulence [23,25]. DspA/E, a virulence factor, is required for pathogenesis of *E. amylovora* [26,27]. *Erwinia avrRpt2*_Ea_ exhibits homology to AvrRpt2 of *Pseudomonas syringae* pv. *tomato*, and is also a known virulence factor [25]; whereas *hopC1* does not contribute to virulence when deleted [23]. Eop1 and Eop3 are YopJ and HopX homologs, respectively, and their role in virulence remains unknown [22,28].

Though studies in other plant pathogenic bacteria have begun to elucidate how type III effectors modulate plant susceptibility and promote bacterial growth and dissemination, effector function in *Erwinia* species is not well studied. Both DspE and HrpN are found to be involved in causing cell death and callose deposition in apple [29,30]. Two recent reports have identified potential host targets for DspE and HrpN [31,32], but the exact molecular mechanism is not well understood. Our recent functional genomic studies using an apple microarray may provide a first glimpse of host reaction to early pathogen infection [33], which could serve as a bridge to further understand *Erwinia*-host plant interaction.

Amylovoran, another major virulence factor, may function in plugging plant vascular tissues, suppressing plant basal defenses, and most importantly, in biofilm formation [34,35]. In *E. amylovora*, 12 amylovoran biosynthetic genes are encoded by the *ams* operon, which is directly regulated by the Rcs phosphorelay system [35,36]. It has been demonstrated that the RcsBCD two-component system is essential for virulence [35,37]. In addition, *in vivo* gene expression technology has identified several two-component systems to be induced during infection of host tissue in *E. amylovora* [23], and genome-wide systematic knockout experiment has demonstrated that four groups of two-component system mutants exhibit varying levels of amylovoran production *in vitro* [37]. These findings suggest that two-component systems in *E. amylovora* play a major role in regulating amylovoran production [37]. Currently, results from our functional genomic studies using whole-genome microarray have suggested that two-component systems may form a gene regulatory network governing the production of amylovoran in *E. amylovora* (Zhao, unpublished).

Natural isolates of *E. amylovora* from North America and Europe have been found to exhibit differential virulence on host apple plants [2,3]. A positive correlation between bacterial virulence on relatively susceptible genotypes, such as Golden Delicious, and the expression/production of major virulence factors such as HrpL, DspE and amylovoran in *E. amylovora* strains has recently been demonstrated [3]. These findings indicate that, although *E. amylovora* as a whole is a genetically homogeneous pathogen [14], the pathogen among Spiraeoideae strains may adapt to different hosts, thus maintaining a population capable of eliciting different levels of diseases on different host plants of varying levels of resistance. However, why some *E. amylovora* strains such as BBA2158 can only infect *Rubus* plant remains elusive. A recent study suggests that the effector Eop1 could act as a host specificity determinant [28]. Furthermore, it has been proposed that the structure of amylovoran, which is known to differ between apple and *Rubus*-infecting *E. amylovora* strains as well as *E. pyrifoliae* [38], may also play a role in host specificity. Indeed, several amylovoran biosynthesis genes in the *ams* operon are very diverse between these *Erwinia* strains, including *amsCDE*, indicating the substrate or specificity of these amylovoran biosynthetic proteins could be different [39]. Furthermore, effectors such as *eop2*, *hopC1* and *avrRpt2* are present in *E. amylovora* strains, but not in *E. pyrifoliae* strains, indicating these effectors may also contribute to host specificity of *E. amylovora* and *E. pyrifoliae*. Another virulence factor in *E. amylovora* is the exopolysaccharide levan; however, the levansucrase gene (*lsc*) is absent in the genome of *E. pyrifoliae* strains. These direct or indirect evidences suggest that host specificity determinants may be very complex (Table 4). It is tempting to postulate that virulence factors act alone or in combination as well as interact with host factors could all contribute to this natural phenomenon.

**Table 4:** Virulence-associated traits and their distribution in *E. amylovora* and related *Erwinia* species.

**Traits**	***E. amylovora***			***E. pyrifoliae***			***E. tasmaniensis***	***E. billingae***
	CFBP 1430	ATCC 49946	BAA 2158	DSM 12163	EP 1/96	Ejp 617	Et1/99	Eb661
**T3SS PAI1**	+	+	+	+	+	+	+ **(P)**	-
**T3SS PAI2**	+	+	+	+	+	+	+	-
**T3SS PAI3**	+	+	+	-	-	-	+ **(P)**	-
**Flagella 1 (S)**	+	+	+	+	+	+	+	+
**Flagella2 (C)**	+	+	+	+	+	+	-	-
**Amylovoran biosynthesis** *	+	+	+	+	+	+	+**(E)**	+**(E)**
**Levansucrase (*lsc*)**	+	+	+	-	-	-	+	-
**Protease A (*prtADEF*)**	+	+	+	-	-	-	-	-
***eop2*, *hopC1*, *avrRpt2***	+	+	+	-	-	-	-	-
***eop1*** **	+	+	+	+	+	+	+	-

* some genes are very diverse such as *amsCDE*, (E): In Et1/99 and Eb661, the *amsE* gene is missing, but additional genes present [6,7]; (P): partial; (S): separated; (C): clustered;

** sequence diversification found in different species.

Analyses of the complete genome sequences of *E. amylovora* and related strains have revealed two additional non-flagellar T3SS PAIs (PAI2 and PAI3) and two flagellar T3SS systems (Fla1 and Fla2) (Table 4) [20]. Phylogenetic tree reconstructed based on the HrcV or InvA protein sequences for all copies from *Erwinia* species can divide the non-flagellar T3SSs into at least five groups. As expected, the PAI1 belongs to the Hrp1group, whereas PAI2 and PAI3 belong to Inv/Mxi/Spa group [40]. Interestingly, PAI2 and PAI3, which have a significantly lower G+C content, are clustered together and closely related to those of *Sodalis glossinidius*. In addition, phylogenetic tree is also constructed based on concatenation of 14 conserved flagellar proteins, which reveals that both Fla1 and Fla2 are clustered with enterobacteria, indicating that these flagellar systems may be originated from enterobacteria [40]. However, the Fla1 system is much closer to the phylogeny of species trees than that of Fla2, which is also closely related to those of *S. glossinidius* [40]. These findings suggest that PAI2, PAI3 and Fla2 may be acquired from a similar source by horizontal gene transfer.

Genetic analyses indicate that both PAI2 and PAI3 appear non-functional in the virulence of *E. amylovora* [20], however, genes on the two PAIs are expressed in rich medium [41], which is unique to plant pathogens, indicating that the two PAIs may play a role during interaction with other hosts such as insects. Comparative genomic analyses with other related *Erwinia* species indicate that most T3SSs are present in *E. pyrifoliae* and *E. tasmaniensis*, with the exception of PAI3 and Fla2, but not in *E. billingae* (Table 4). Determining the function of these additional islands in *E. amylovora* may provide us with clues as whether they may have a role in host specificity or during interaction with insect vectors, which remains to be elucidated.

## 5. Conclusion and Future Perspectives

In summary, genome sequences of four species in the genus *Erwinia* have provided us with the genetic composition of these conserved erwinias. Comparative genomic analyses have helped us to draw preliminary conclusions about the evolution and the classification of *Erwinia* species. However, the host specificity and differential virulence phenomenon of *Erwinia* strains is still not completely understood. Fully understanding the pathogen, its virulence mechanism and host specificity is very promising as whole genome sequencing and functional genomic studies are powerful hypothesis generators. With the advances of technologies and multidisciplinary collaboration, future work should address questions, to mention just a few: what are the functions of the PAI2 and PAI3 during interaction with insect vectors? What is the function of type VI secretion systems in erwinias, if there is any? What is the molecular mechanism of effector protein function such as DspE/A when they are translocated inside plant cells? Reconstructing the gene regulatory network of amylovoran biosynthesis using functional genomics tools such as microarray and computer modeling is also vital. We expect that there will be tremendous progress in the next decade or so in studying fire blight and related plant diseases, which will ultimately lead to the development of environmentally sound disease management strategies.
